# Kinematics of Gait: New Method for Angle Estimation Based on Accelerometers

**DOI:** 10.3390/s111110571

**Published:** 2011-11-07

**Authors:** Milica D. Djurić-Jovičić, Nenad S. Jovičić, Dejan B. Popović

**Affiliations:** 1 School of Electrical Engineering, University of Belgrade, Bulevar kralja Aleksandra 73, Belgrade, Serbia; E-Mails: nenad@etf.rs (N.S.J.); dbp@etf.rs (D.B.P.); 2 Tecnalia Serbia, Vladetina 13/6, Belgrade, Serbia; 3 Center for Sensory Motor Interaction, Aalborg University, Fredrik Bajers Vej 7, Aalborg, Denmark

**Keywords:** accelerometers, ambulatory system, angles, gait assessment

## Abstract

A new method for estimation of angles of leg segments and joints, which uses accelerometer arrays attached to body segments, is described. An array consists of two accelerometers mounted on a rigid rod. The absolute angle of each body segment was determined by band pass filtering of the differences between signals from parallel axes from two accelerometers mounted on the same rod. Joint angles were evaluated by subtracting absolute angles of the neighboring segments. This method eliminates the need for double integration as well as the drift typical for double integration. The efficiency of the algorithm is illustrated by experimental results involving healthy subjects who walked on a treadmill at various speeds, ranging between 0.15 m/s and 2.0 m/s. The validation was performed by comparing the estimated joint angles with the joint angles measured with flexible goniometers. The discrepancies were assessed by the differences between the two sets of data (obtained to be below 6 degrees) and by the Pearson correlation coefficient (greater than 0.97 for the knee angle and greater than 0.85 for the ankle angle).

## 1. Introduction

Gait analysis is important for objective assessment of the effects of rehabilitation interventions. The most accurate systems for gait analysis are camera-based systems with reflective markers [1]. These systems acquire spatial movement (3D) of many markers positioned on the body, while a software outputs the joint angles and/or other gait parameters. However, camera-based systems require a dedicated laboratory and limit the length of the analyzed walking distances. Gait laboratories also use force platforms to measure the ground reaction forces which typically record from only one or two steps in the middle of the gait sequence. The platforms are 60 × 60 cm, so that aiming for the platforms hinders the subjects’ natural gait patterns. The alternative to camera-based systems are ultrasound systems [2] and magnetic tracking systems [3], which allow complete 3D kinematic analysis of human movements.

Over the last decade, many gait analysis systems using non-traditional methods have been developed. These systems, for example, use laser technology or measure near-body air flow [4,5] in order to estimate kinematics and spatial gait parameters. Also, electronic carpets or wearable force sensors are used for estimation of ground reaction forces, centre of pressure, and temporal gait parameters [6,7]. Since there is often a need for gait recording in various environments, portable body-mounted systems are preferred [8,9].

Portable body-mounted systems allow data acquisition from many steps. The portable systems for kinematics data acquisition directly measure joint angles, or they can record accelerations or angular velocities of the body segments that carry the sensors. Measurement of joint angles can be done with various electrogoniometers [9–11]. Particularly convenient are flexible goniometers, which measure the relative angle between two small blocks that are fixed to the body segments (e.g., Biometrics flexible Penny & Giles sensors). The advantages of flexible goniometers are: their output is directly proportional to the angle and their mounting is simpler compared to some other measurement systems. However, they are not sufficiently robust for daily clinical usage.

An alternative to goniometers, offered by the progress made in micro-electromechanical systems (MEMS), is the use of accelerometers and gyroscopes. The advantages of these sensors include their small size and robustness when compared with goniometers. However, the disadvantages of the accelerometers (and gyroscopes) are computational problems for determining the angles [12–15].

Accelerometers are used for long-term monitoring of human movements, for assessment of energy expenditure, physical activity, postural sway, fall detection, postural orientation, activity classification and estimation of temporal gait parameters [16–21]. However, only a few papers report using only accelerometers for angle estimation, or position and orientation estimation. In most papers some additional types of sensors are included (gyroscopes, magnetometers, *etc.*) [22–25].

One method to calculate the angle is to double integrate the measured angular acceleration. However, the double integration leads to a pronounced drift [13,14]. Several techniques have been presented in literature for minimization of this drift. For example, Morris [26] identified the beginning and the end of each walking cycle and made the signals at the beginning and the end of the cycle equal. Tong *et al.* [27] applied a low cut high pass filter on the shank and thigh inclination angle signals. However, these methods also removed the static and low-frequency information about the angles and they cannot be applied to real-time processing.

The other method for estimation of joint angles from the measured accelerations is the estimation of the inclination angles between the segments (sensor) and the vertical, followed by the subtraction of the angles for neighboring body segments. The results are acceptable only if the segment accelerations are small compared to the gravity [28].

Adding Kalman filtering to the integration procedure decreases the drift and provides for real-time applications, but it requires calibration and data from other sensors (accelerometers, gyroscopes, and magnetic sensors in most cases) for error minimization, as well as noise statistics and good probabilistic models [29–31]. These algorithms can be applied in real-time and seem to give excellent accuracy for motions which exhibit lower accelerations than the leg segments, and which are not exposed to impacts like those of heel contacts. For the lower extremities, the performance of the Kalman filter is considerably reduced when measuring the orientation angles of segments that move fast [28]. Inertial sensors that consist of accelerometers, gyroscopes, and magnetometers, along with Kalman filtering, allow a good accuracy for estimation of lower limb angles [32]. However, good accuracy of angle estimation can also be achieved using fewer sensors and much simpler algorithms that are not sensitive to the presence of metals and ferromagnetic materials such as those that comprise magnetometers [23].

Willemsen *et al.* [32] developed a technique to estimate joint angles without integration. This method is based on comparison of weighted accelerations of the joint (e.g., knee or ankle) obtained from two accelerometer pairs mounted on two adjacent segments of the leg. The method requires adequate low-pass filtering, which introduces a delay and to a certain extent hinders the real-time applicability. Further, the accelerometer pairs need to be precisely oriented, so that their axes intersect at the joint, which is very difficult to achieve considering that the human joints are polycentric. Also, the distances between sensors and the joints are required for computation.

We have developed an accurate, yet simple method and instrumentation for estimation of absolute segment and joint angles during the gait (assuming kinematics in the sagittal plane) which minimizes the effects of drift. The proposed system is based only on accelerometer sensors, which is advantageous because their calibration is static and less complex than the dynamic calibration required for gyroscopes. Additional motivation for this paper was the “bad reputation” of accelerometers due to the pronounced drift. We wanted to investigate if it is possible to use only accelerometers for angle estimations and evaluate the precision of the results.

## 2. Experimental Section

### 2.1. Sensor System

The acquisition system that we developed for gait analysis is designed as a distributed wireless sensor network. A set of battery powered sensor nodes is placed on the subject, one sensor node for each leg segment of both legs. Sensor nodes establish communication with the coordinator node through a low power 2.4 GHz wireless communication link. The coordinator node is connected using a USB interface to the computer. Wireless communication is bidirectional, with a coordinator node acting as a master, and the sensor nodes as slaves. The coordinator node manages network traffic and the USB connection with the computer. Data streams from the sensor nodes are synchronized and the system operates with a 100 Hz sampling rate.

Sensor nodes are realized as a sandwich structure of processor and sensor board with a Li-ion battery placed between the boards. The compact size design of sensor nodes, with dimensions 70 × 25 × 15 mm and 27 grams weight, enables comfortable wearing and does not hinder the subject’s movements. Hardware design is based on the Texas Instrument’s CC2430 microcontroller, which integrates a RF front end and a 8051 core in the same case. Standard microcontroller peripherals enable interfacing to analog and digital sensors, and different sensor boards can be combined with the same processor board.

**Figure 1.**
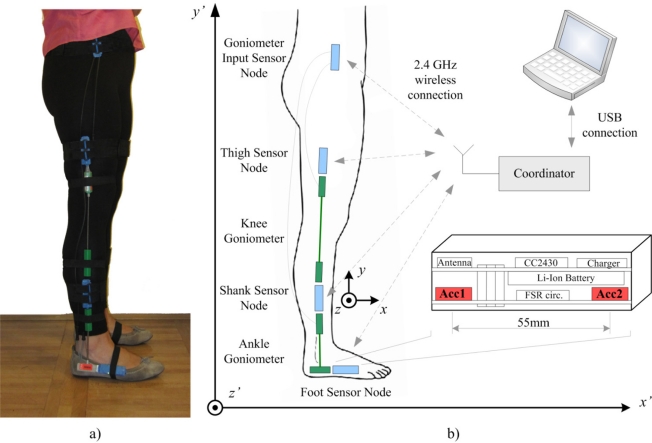
Setup of the sensor system. **(a)** photo of the sensors mounted on the body during the gait analysis, **(b)** schematic of the system configuration with the coordinate systems.

In the configuration used in this research, the sensor board comprises two high performance 12-bit digital accelerometers LIS3LV02 (SGS-Thomson Microelectronics, USA). The range of the sensors is either ±2 g or ±6 g, which can be selected in the acquisition software. Accelerometers are aligned to *y* axes with distance of 55 mm between centers. This configuration requires the clinician only to fix the sensor array along the body segment, approximately at the mid section of lateral side of leg (Figure 1).

Goniometers were attached to the leg segments by using double sided adhesive tape and secured with elastic bands with Velcro endings, mounted over the sensors and around the leg segment. Sensor nodes were placed in custom made tight sensor node-size elastic pockets placed on elastic bands with Velcro at their ends.

The custom-designed software, created in CVI (LabWindows, National Instruments, USA), is used for online monitoring and storing of the acquired data.

### 2.2. Algorithm

**Figure 2.**
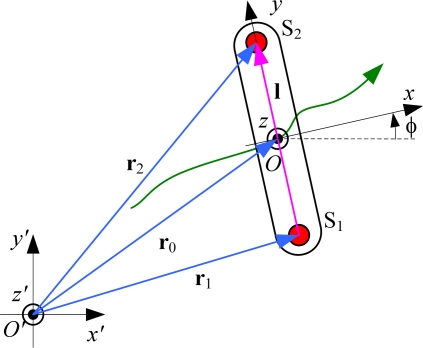
Rod with two accelerometers and the coordinate system for analysis of movement in the sagittal plane.

The mechanics of importance for the analysis considers two sensors (denoted by S_1_ and S_2_), which are mounted on a rigid rod (Figure 2). The distance between the sensors is *l*. The rod is freely moving with respect to the fixed global coordinate system (*O*′*x*′*y*′*z*′), shown in Figures 1(b) and 2. The axis *x*′ of the global coordinate system is walking direction, and the axis *y*′ is vertical. The center of the rod (*O*) is determined by the position vector $\vec{O^{\prime }O}=r_{0}(t)$.

To analyze the movement in the sagittal plane, we consider the case when the rod moves in the *O*′*x*′*y*′ plane (2D model). We define the vector **l**, which connects the centroids of the two sensors. The positions of the accelerometers are **r**_1_ = **r**_0_ −**l** / 2 and **r**_2_ = **r**_0_ + **l** / 2, respectively.

Each accelerometer measures the two Cartesian components of the acceleration vector, with respect to the local coordinate system *Oxy* attached to the rod. The equivalent accelerations measured by the two sensors are:

(1) $$a_{1}=\frac{d^{2}r_{1}}{dt^{2}}-g={\ddot{r}}_{0}-\frac{\ddot{l}}{2}-g$$

and:

(2) $$a_{2}=\frac{d^{2}r_{2}}{dt^{2}}-g={\ddot{r}}_{0}+\frac{\ddot{l}}{2}-g$$

where **g** is the gravity acceleration.

The difference of the signals from these two sensors is proportional to the amplitude of the vector **a**_1_ – **a**_2_ = −**l̈**. In this way, we cancel out the influence of the movement of the rod centroid and of the gravity, and retain information only about the changes of the vector **l**. The second derivative of the vector **l** is:

(3) $$\ddot{l}=l\ddot{\varphi }u_{0}-l{\dot{\varphi }}^{2}l_{0}=l\alpha i_{x}-l\omega^{2}i_{y},$$

where φ is the angle between the axes *x* and *x*′, ω and α are the absolute angular velocity and angular acceleration of the rod, respectively, **i***_x_*, **i***_y_*, and **i***_z_* are the unit vectors of the *x*, *y*, and *z* axes of the rod, respectively, *l* = |**l**|, **l**_0_ = **l** / *l* = **i***_y_*, and **u**_0_ = **i***_z_* × **l**_0_ (where **i***_z_* is the unit vector of the axis of rotation).

The difference of the outputs from the accelerometers in the direction along the rod axis (Δ*a_y_*) is proportional to the square of the angular velocity, and the difference between the outputs from the accelerometers in the perpendicular direction (Δ*a_x_*) is proportional to the angular acceleration of the segment. The proportionality coefficient is equal to the distance between the centers of the accelerometers. In this way we eliminated the gravity component from the signal, and eliminated the need for precise positioning of the rod on the body segment and calibration of the system.

**Figure 3.**
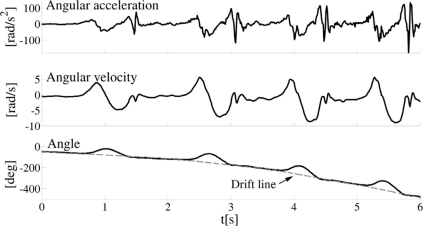
Joint angle (bottom panel) and angular velocity (middle panel) obtained by numerical integration of the measured acceleration of the segment (top panel). Dashed line envelope on the bottom panel is fitted through the points where the knee should be fully extended with zero degree joint angle. However, due to the integration drift, instead of remaining approximately constant, this line has a parabolic shape.

As explained in the introduction, one of the main problems with accelerometers is significant drift after integration, whether the integration is performed numerically or by means of analog integrators. A characteristic example of the drift, which resulted even with carefully calibrated accelerometers, is presented in Figure 3.

We introduce a method for estimation of the joint angles based on digital filtering. In order to explain the method, we use the frequency domain. According to the Laplace transform, the integration in the time domain corresponds to multiplication by 1/*s* in the frequency domain, and the double integration corresponds to multiplication by 1/*s*^2^, where *s* is the complex frequency. On the frequency axis (*i.e.*, in the Fourier-transform domain, where *s* = jω), this corresponds to multiplication by 1/(jω)^2^ = −1/ω^2^. Hence, we use a second-order low-pass filter, which mimics this multiplication. Further in the paper, we shall write − 1/ω^2^ instead of 1/*s*^2^, because we wish to emphasize the fact that this multiplicative term is purely real (although negative).

Without the loss of generality, we can assume that the signal Δ*a_x_* (*t*) /*l* is nearly periodic. Hence, it has pronounced spectral components at *f_i_* = *i* /*T*, *i* = 1,2,..., where *T* is the stride period. All relevant spectral components of Δ*a_x_* (*t*) /*l* should be in the roll-off region of the filter, where its transfer function is proportional to −1/ω^2^. For example, the transfer function of the second-order Butterworth filter is:

(4) $$B_{2}(s)=\frac{1}{{\left(\frac{s}{\omega_{0}}\right)}^{2}+\sqrt{2}\left(\frac{s}{\omega_{0}}\right)+1}$$

where ω_0_ = 2π*f*_0_ is the cutoff angular frequency. On the imaginary axis, when | *s* |>> ω_0_, we get:

(5) $$B_{2}(s)\approx \frac{\omega_{0}^{2}}{s^{2}}=-\frac{\omega_{0}^{2}}{\omega^{2}}$$

In order to approximate the double integration, we pass the signal through the filter and divide the output by $\omega_{0}^{2}$.

**Figure 4.**
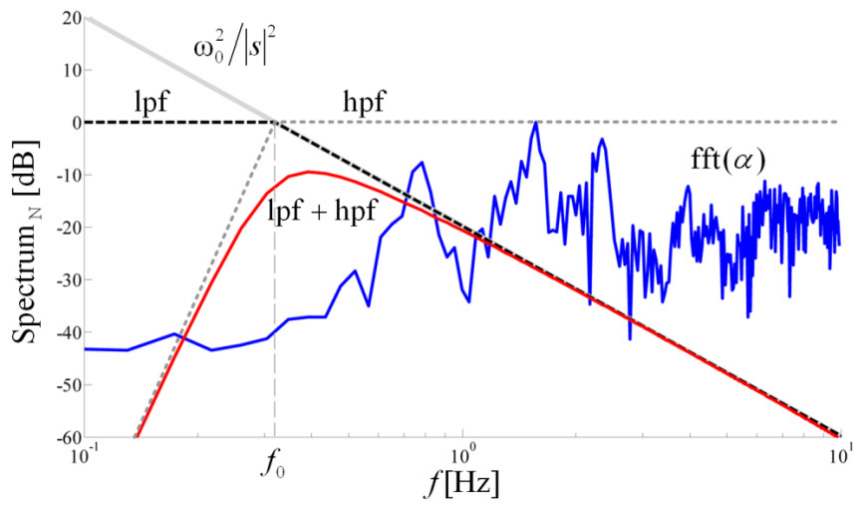
Normalized spectrum of the angular acceleration for knee angle (shown in Figure 3) and magnitudes of the function $\omega_{0}^{2}/{\left|s\right|}^{2}$, transfer function of a second-order Butterworth low-pass filter (lpf), and function of this low-pass filter combined with a high-pass filter (lpf + hpf).

The filter is, however, dispersive. Various spectral components have various delays and the filtered signal will only barely resemble the actual function φ(*t*). A zero-delay filter can be obtained by bi-directional filtering, using the filtfilt function in Matlab. First, the signal is filtered in the forward direction. Then, the filtered sequence is reversed and run back through the filter. This procedure results in a real and positive transfer function (zero-phase distortion and zero group delay), whose order corresponds to double the filter order. For example, if we use the first-order Butterworth filter, whose transfer function is *B*_1_(*s*) = 1/(*s* /ω_0_ + 1), the result of the bidirectional filtering is the transfer function $1/({\left(\omega /\omega_{0}\right)}^{2}+1)\approx \omega_{0}^{2}/\omega^{2}$ when ω >> ω_0_. Hence, to obtain the −1/ω^2^ transfer function, we use the first-order Butterworth filter with the filtfilt function and divide the result by $-\omega_{0}^{2}$. Figure 4 shows the normalized spectrum of the angular acceleration, along with the $\omega_{0}^{2}/{\left|s\right|}^{2}$ function, and the magnitude of the transfer characteristic of the low-pas filter (Bode plot), low-pass filter combined with a high-pass filter obtained by the filtfilt function.

**Figure 5.**
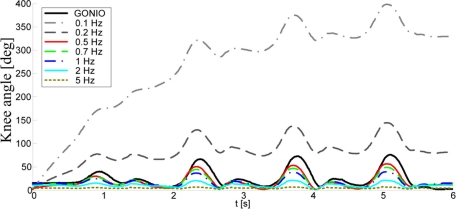
Influence of *f*_0_ on angle estimation: filtering for several cutoff frequencies compared with angle acquired from goniometers.

The choice of *f*_0_ followed the heuristics (Figure 5). If *f*_0_ is too low, joint angles exhibit drifting similar to the numerical integration. On the other hand, in order to keep the spectral components in the roll-off region of the filter, the condition *f*_0_ < *f_gc_* = 1/*T* should be fulfilled. If this condition is not respected and *f*_0_ is taken to be higher than *f_gc_* (where *f_gc_* is the gait cycle frequency), one or more spectral components are within the pass band of the filter, where the transfer function of the filter is approximately constant and close to 1. Increasing further the filter bandwidth, *i.e.*, increasing *f*_0_, these components are not affected by the filter. However, their magnitudes are divided by $\omega_{0}^{2}$, so that the level of these components is reduced, and the result is distorted. On the contrary, the magnitudes of the spectral components that are in the roll-off region of the filter are insensitive to the modifications of *f*_0_. Since filtering should replace the integration, all relevant spectral components of the gait should be in the roll-off region of the filter, *i.e.*, well above the cutoff frequency of the filter. By comparing the filter amplitude characteristic, which is the modulus of (4), and $\omega_{0}^{2}/\omega^{2}$, it can be verified that the error between these two functions is less than 1 dB for spectral components that are above two times the cutoff frequency of the filter. Similarly, the error is less than 0.5 dB for spectral components above three times the cutoff frequency. Hence, the cutoff frequency *f*_0_ of the filters is determined so that the lowest relevant spectral component of the signal is positioned between 2 *f*_0_ and 3 *f*_0_.

The drift can be additionally reduced if a high-pass filter is used in conjunction with the low-pass filter. The cutoff angular frequency of the high-pass filter should be below ω_0_, so that the major role of the low-pass filter is not affected. The order of the high-pass filter can be selected as an additional parameter to help keep the drift under control.

As an example, Figure 4 shows the magnitude of the transfer characteristic of the combined low-pas filter and a high-pass filter of 8th order, which is an actual filter used in computations in this paper. Since filtering distorts the DC level, we restore this information through the self-calibration in the following way. Before gait initiation, the subject needs to remain standing still (and sensors immobile) for at least two seconds. During this interval, the initial conditions are determined for each pair of accelerometers by using them as inclinometers.

The procedure of approximating the double integration can be applied both for reconstruction of absolute angles (the angles between the rod axes and the vertical axis of the fixed coordinate system) and the reconstruction of the joint angles. For example, the knee angle is obtained directly from the difference:

(6) $$\Delta a_{x\;\text{thigh}}(t)/l-\Delta a_{x\;\text{shank}}(t)/l$$

by performing the bi-directional filtering, and divide the result by $-\omega_{0}^{2}$. An analogous procedure is performed for the ankle angle.

### 2.3. Experiments

The algorithm was tested on 27 healthy subjects walking on the ground at their natural pace. In order to provide a more systematic validation, we additionally recorded 10 subjects (age: 26 ± 1.5 mean ± SD) walking at various speeds on treadmills (Life Fitness 9500HR and Panatta Advance Lux 1AD003), whose results are presented in this paper.

Four trials per subject were recorded. Besides walking, recording sequence also included standing still for at least 2 s before and after each walking sequence, which was used for self-calibration and checking. Subjects were walking with various velocities on a treadmill, starting from 0.15 m/s and incremented by 0.05 m/s up to 2 m/s. As the reference system for this study, we used SG110 and SG150 flexible goniometers with the joint angle units for signal conditioning (Biometrics, Gwent, UK). Goniometers were mounted on the lateral side of the leg (at the ankle and knee joints) following the instructions of the manufacturer. Simultaneously, the sessions were recorded with a video camera for later analysis.

### 2.4. Processing of Measured Data

Based on the recorded accelerometer data, the joint angles were estimated by the proposed algorithm. Joint angles recorded by goniometers were also computed. The accuracy of our algorithm was evaluated in terms of the root-mean-square error (RMSE) as well as the Pearson’s correlation coefficients (PCCs) between the goniometer results and angles provided by the proposed method. RMSE is expressed in degrees. PCC values range between −1 and 1, where 1 represents the best possible similarity between the two sets of angles (identical shapes). The first and the last stride were excluded from each trial, and comparison between goniometer signals and angles provided by our method was done on the remaining sequence. The data processing was done offline using Matlab 7.5 (Mathworks, USA).

## 3. Results

**Figure 6.**
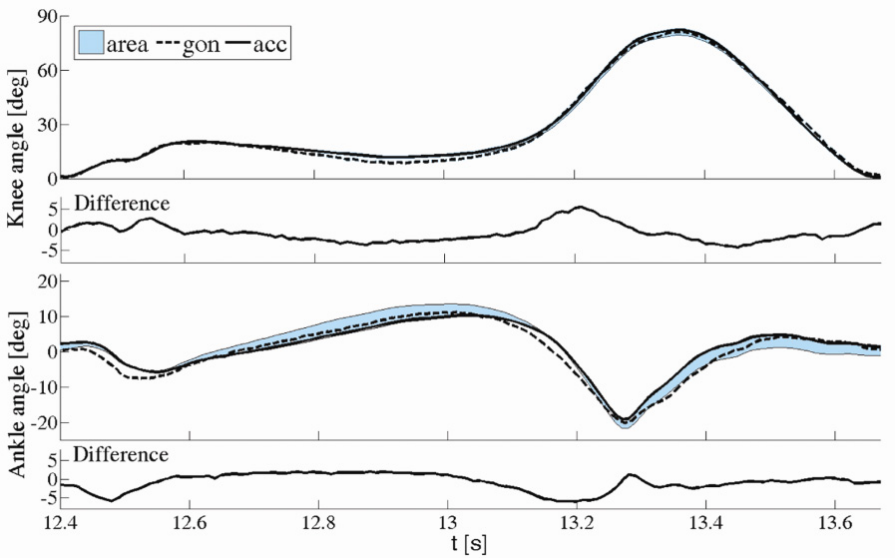
Knee and ankle joint angles measured with goniometers and estimated from accelerometers. Thick lines represent angles from goniometers (dashed line) and accelerometers (solid line) for optimal filter frequency. Blue areas show the range of angle values estimated from accelerometers when filtered with various frequencies in the range [*f_gc_* /3, *f_gc_* /2].

Two typical examples for the knee and ankle angles are shown in Figure 6. The error was defined as the difference between the angles obtained by the proposed method and the angles obtained from goniometers.

Based on the results obtained from treadmill recordings, the cutoff frequency for the knee angles should be in the range [*f_gc_* /3, *f_gc_* /2], where *f_gc_* is the gait cycle frequency. The blue area in Figure 6 shows the family of curves estimated by our method when filtered with various frequencies in the range [*f_gc_* /3, *f_gc_* /2].

**Figure 7.**
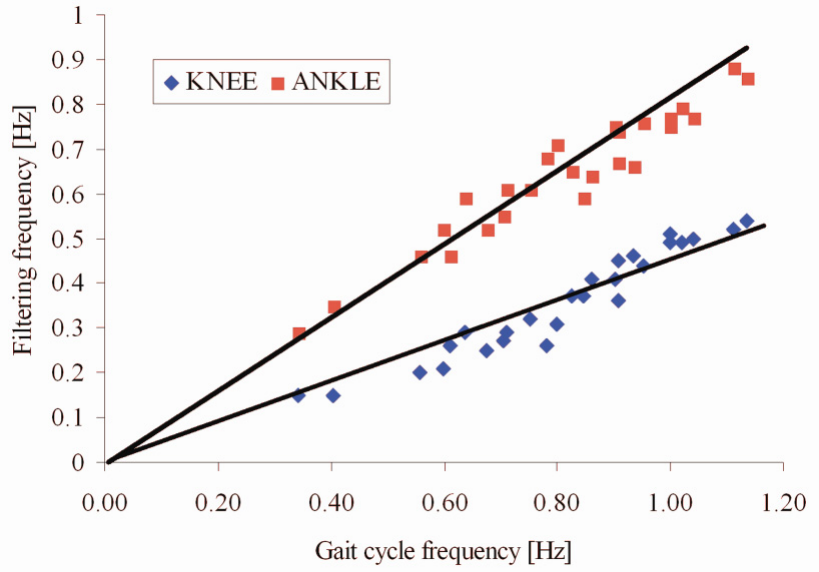
Optimal filtering frequencies for knee and ankle angles *versus* gait cycle frequency.

Figure 7 shows the optimal filtering frequency *versus* gait cycle frequency. The squares represent optimal points obtained by maximizing Pearson’s correlation coefficient and minimizing RMSE between our results and the angles obtained by goniometers for each walking trial (different gait velocity). The straight lines are obtained by fitting these data. It is obvious from Figure 7 that signals for the estimation of the ankle angles should be filtered with about two times higher cutoff frequency than the signals used for the estimation of the knee angles. These findings are in agreement with the theoretical background from the previous section.

The higher cutoff frequency of the filter for the ankle angle can be used because the spectrum of the ankle angle has a very pronounced second harmonic. This is convenient, because the influence of the drift is further suppressed.

**Figure 8.**
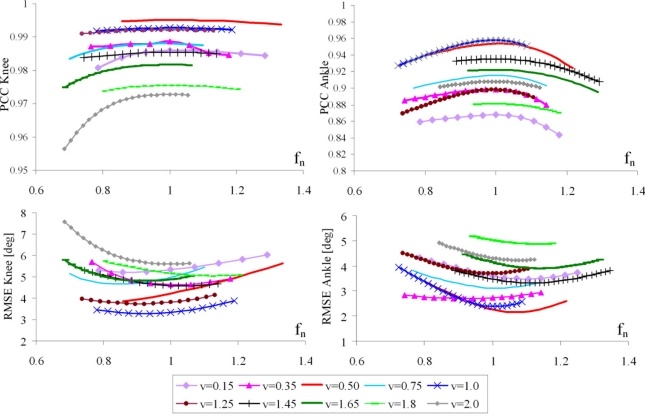
Pearson’s correlation coefficient (PCC) and RMSE between goniometers and estimated angles, for knee and ankle angles, *vs.* filtering frequency in the range [*f_gc_* /3, *f_gc_* /2]. The normalization is with respect to the optimal frequency. Each curve corresponds to different gait velocity (in m/s).

Using the proposed algorithm, for each subject and each trial, we evaluated the PCC and RMSE values between the angles estimated by our method and goniometer outputs. Figure 8 shows the PCC and RMSE, respectively, as a function of normalized frequency, for various velocities. Although we recorded velocities from 0.15 to 0.2 m/s in the steps of 0.05 m/s, Figure 8 shows results for a subset of the recorded velocities.

**Figure 9.**
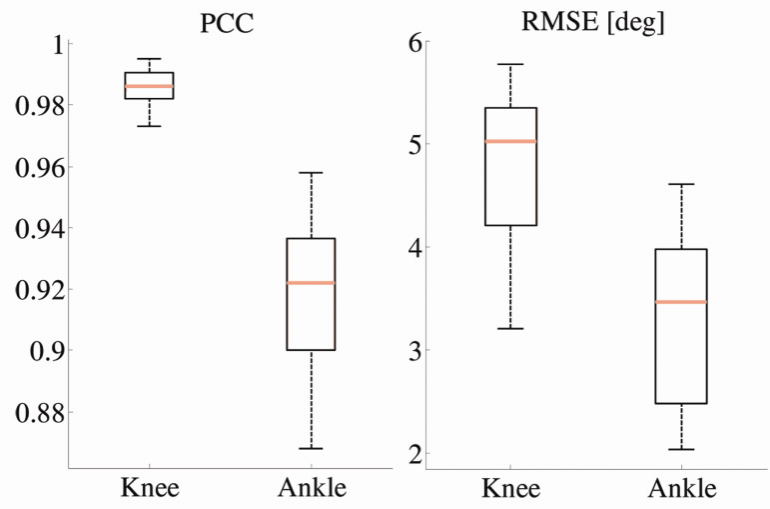
Boxplots presenting comparison between joint angles (for knee and ankle angles) calculated from accelerometers and goniometers. Left: Pearson’s correlation coefficient (PCC). Right: root-mean-square error (RMSE).

As shown in Figure 8, all RMSE curves have broad minima, which are, on average about 1. These results are in accordance with PCC curves, confirming that the optimum normalized cutoff frequency is 1. Cumulative results for all walking trials and all subjects are presented in Figure 9.

## 4. Discussion and Conclusions

The presented results are based on a model which assumes that the lower limbs move in the sagittal plane. For healthy subjects, this 2D model has proven to be sufficient, because the sagittal plane is the plane where the majority of movement takes place. Generally, for clinical applications, the proposed method provides an acceptable accuracy for angles and high correlation coefficients with the measurements obtained from goniometers.

In particular, PCCs for the knee angle are higher than 0.97 and RMSE is within 6° for the angle values. Further, our results showed that a 5° RMSE is obtained for walking speeds in the frequency range *f_gc_* ∈ [0.35, 1.15]. This corresponds to all velocity curves in Figure 8 except for v = 0.15, 1.8, and 2.0 m/s (the slowest and fastest recorded walking). Regarding the ankle angles, PCCs are slightly lower and in the range from 0.85 to 0.97, while RMSE is from 2° to 4.7°.

For both estimated joint angles (except for the extreme velocities for the knee angle), this error is in the range of 5° mean error limit accepted by the American Medical Association to consider the measurements reliable for the evaluation of movement impairments in a clinical context [9]. The accuracy of our simple system is comparable to the accuracy demonstrated in plots presented in [25], which were obtained by much more complex hardware and software.

Joint angles were determined by subtracting the absolute angles of the neighboring leg segments. The error of our method was estimated based on joint angles and includes errors from the two segments. In this way, the total error of the joint angle estimation is different than the error of the absolute angles. Hence, comparison of the absolute angles with a camera system would be more appropriate for validation of the proposed method. However, such a comparison was not possible in our experiments because the treadmill would present a visual obstacle between cameras and markers. Since our main goal was to investigate how our method performs for various gait velocities, the treadmill was the important part of the experiments. Therefore, we selected goniometers as the reference system, which have ±2° accuracy and 1° repeatability [33]. Although electrogoniometers are prone to errors due to potential misalignment with the femur and tibia segment in the sagittal plane, this does not affect the validation of our method since we secured sensor units to be aligned with goniometer blocks.

Skin motion artifacts cause errors to all body-fixed sensors. Sensors placed on thighs are more susceptible to skin and soft tissue related motion, because the majority of femur is concealed by a substantial amount of soft tissue. However, the errors that we report here are much smaller than errors due to rod misalignment in the procedure proposed by Willemsen [32].

Another limit for this algorithm is the speed of the subject’s gait. As it can be seen from Figures 7 and 8, if the gait is very slow, the quality of our method decreases. This is due to very low angular accelerations, whose major component comes from the impacts. This suggests that for a very slow walk, e.g., for subjects with high levels of disability, the quality of the angle estimation may not be acceptable. However, for very slow gait, accelerometers can be used as inclinometers and angles can be successfully estimated in this way [17].

Our method does not need information about the distances to joint centers or distances between sensor rods placed on different segments, which is one of the benefits of this algorithm. The only condition for mounting the sensors is that they follow the segment line (to be aligned with a line connecting adjacent joints, viz. hip and knee, knee and ankle, and along the foot and parallel to the ground).

This algorithm could be used not only for level walking, but also for estimation of angles during slope walking, stair climbing, or any other rhythmical (periodic) leg movements. It can also be used for estimation of other segment and joint angles, as long as the movements are in 2D. However, movements should be fast enough so that the angular acceleration signal is sufficiently above the noise floor. The proposed method is suitable for postprocessing of raw data. However, it can also be included into real-time algorithms to estimate the angles of the leg segments with a delay of one stride.

The proposed method is simple and computationally efficient. We have demonstrated that it yields accurate shapes of the ankle and knee angles. The accuracy of the method is sufficient for quick diagnostics of gait, as well as for applications of gait control.
